# An Energy-Dispersive X-ray Spectroscopy (EDX)- and Tensile Test-Based Investigation of Mechanical Properties and Elemental Analysis of Three Commercial Stainless Steel Orthodontic Archwires

**DOI:** 10.7759/cureus.111173

**Published:** 2026-06-19

**Authors:** Ravisankar Vijayan, Latheef Vadakkepediyakkal

**Affiliations:** 1 Orthodontics and Dentofacial Orthopaedics, Government Dental College, Thrissur, IND; 2 Orthodontics and Dentofacial Orthopaedics, Government Dental College, Kozhikode, IND

**Keywords:** composition, corrosion resistance, deflection, mechanical properties, modulus of elasticity, stainless steel archwire, stiffness, tensile test, ultimate tensile strength, yield strength

## Abstract

Introduction: Stainless steel (SS) continues to be the preferred working archwire during orthodontic retraction. Inter-manufacturer differences and clinical preferences suggest that all brands of the same dimension do not perform identically. Hence, the objective of the study is to compare the elemental composition and mechanical properties of three commercially available SS archwire brands representing different economic tiers in the market: 3M Unitek (Monrovia, CA, USA; high tier), G&H Orthodontics (Franklin, IN, USA; medium tier), and DTech (Pune, India; low tier).

Materials and methods: Seven specimens from each group (Group I: 3M; Group II: GH; Group III: DT) were subjected to scanning electron microscopic (SEM) evaluation coupled with elemental analysis using energy-dispersive X-ray spectroscopy (EDX). They were also subjected to tensile testing using a universal testing machine for evaluating ultimate tensile strength (UTS), yield strength (YS) and modulus of elasticity (E).

Results: SEM evaluation showed variation in carbon concentration across the groups, with the highest in 3M (7.3%), followed by DT (5.5%) and GH (3.9%). UTS and YS were highest for 3M, followed by DT and GH. E was highest for DT, followed by GH and 3M. However, all values were statistically insignificant.

Conclusion: The corrosion resistance of commercially available SS archwires may not be consistent with the expected material properties because of the marked variation in carbon content among brands. 3M might deform less in the oral cavity with better fracture resistance, indicating superior mechanical stability. DT exhibited greater stiffness, making it more useful in cases requiring occlusal plane control. GH demonstrated intermediate performance between the groups.

## Introduction

Stainless steel (SS) archwires have remained popular since their introduction to orthodontics. SS was introduced by Harry Brearley in 1912, introduced into dentistry by Hauptmeyer in 1919 and later into orthodontics by Friel in 1933 [1]. It continues to be the preferred working archwire during orthodontic retraction because of high stiffness, excellent formability, biocompatibility, environmental stability, surface smoothness, and low cost [2]. These properties make them highly suitable for resisting deformation produced by intra-arch, interarch, and extraoral force systems. 

Austenitic SS used in orthodontics contains very low carbon content. The composition reported by Brantley and Eliades [3] is approximately 17-20% chromium, 8-12% nickel, with carbon usually below 0.15%, and the remainder predominantly iron. Likewise, Harvey [4] reported that the carbon content in SS typically ranges from 0.08 to 0.15 wt%. Corrosion resistance of SS is largely due to the presence of chromium in the alloy, which creates a passive layer of oxide at the exposed surfaces [3].

The market is flooded with various brands of SS archwires, and manufacturers often do not explicitly disclose their material properties. Consequently, product selection is frequently influenced by local clinical preferences and practitioner curiosity regarding inter-manufacturer differences [2]. Clinical experience also suggests that not all brands perform identically; some wires of the same dimension appear less rigid and undergo greater deflection under applied forces, thereby influencing the final displacement pattern of the dentition. Therefore, the choice of archwire requires careful evaluation of the clinical requirements together with an appropriate balance between physical properties, cost-effectiveness, and material availability [5].

Accurate evaluation of material properties of orthodontic materials has become increasingly important with the growing emphasis on analytical methods in appliance design. Because SS archwires continue to be used extensively in contemporary orthodontic practice and are likely to remain integral to future treatment mechanics, the present study was undertaken to evaluate the properties of SS archwires from three commonly used manufacturers. The objective of this study was to perform elemental analysis and compare the ultimate tensile strength (UTS), modulus of elasticity (E) and yield strength (YS) of three commercially available SS archwire brands representing different economic tiers in the market: 3M Unitek (Monrovia, CA, USA; high tier), G&H Orthodontics (Franklin, IN, USA; medium tier), and DTech (Pune, India; low tier).

## Materials and methods

Preformed straight-length 0.019 x 0.025-inch SS archwires from three manufacturers - 3M Unitek, G&H Orthodontics, and DTech - were included in the study and divided into three groups: Group I (3M), Group II (G&H), and Group III (DTech). Seven specimens from each group were subjected to elemental analysis and evaluation of UTS, E, and YS.

Elemental analysis 

**Figure 1:**
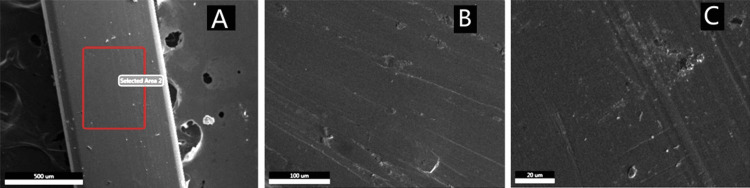
Scanning electron micrograph of one of the wires (3M Unitek, Monrovia, CA, USA). A, 500 µm magnification. B, 100 µm magnification. C, 20 µm magnification.

**Figure 2:**
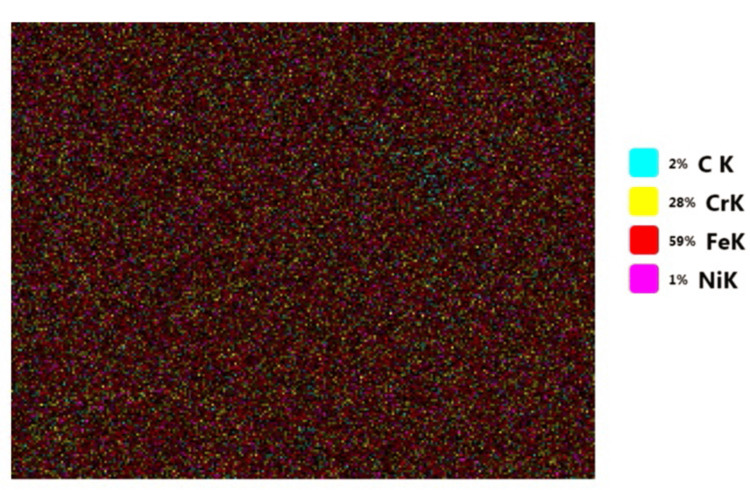
Element overlay of one of the wires. C K, carbon; CrK, chromium; FeK, iron; NiK, nickel.

Specimens from groups I to III were subjected to scanning electron microscopic (SEM) evaluation (Figure 1) coupled with elemental analysis using energy-dispersive X-ray spectroscopy (EDX, or EDS) to assess the compositional characteristics of the SS alloys (Figure 2). The analysis was performed to identify and compare inter-manufacturer variations in elemental composition among the tested archwires. This technique facilitated identification and mapping of elemental distribution at the micro- to nanometre scale, thereby allowing the characterisation of the metallurgical composition of the tested SS archwires. A standardised 100 µm x 100 µm area at 1000x magnification was scanned on the flat surface of each wire specimen, and five randomly selected areas per sample were analysed, with the reported values representing the mean of these measurements. Prior to analysis, specimens were ultrasonically cleaned in 99% ethanol for 10 minutes, rinsed with deionised water, and dried with compressed nitrogen. No conductive coating was applied because the metallic orthodontic alloys were sufficiently conductive for analysis. Carbon and other light elements were evaluated using standard matrix correction algorithms (ZAF/Phi-Rho-Z). An SEM is given in Figure 1 and an EDX elemental overlay is depicted in Figure 2.

UTS, E, and YS

**Figure 3:**
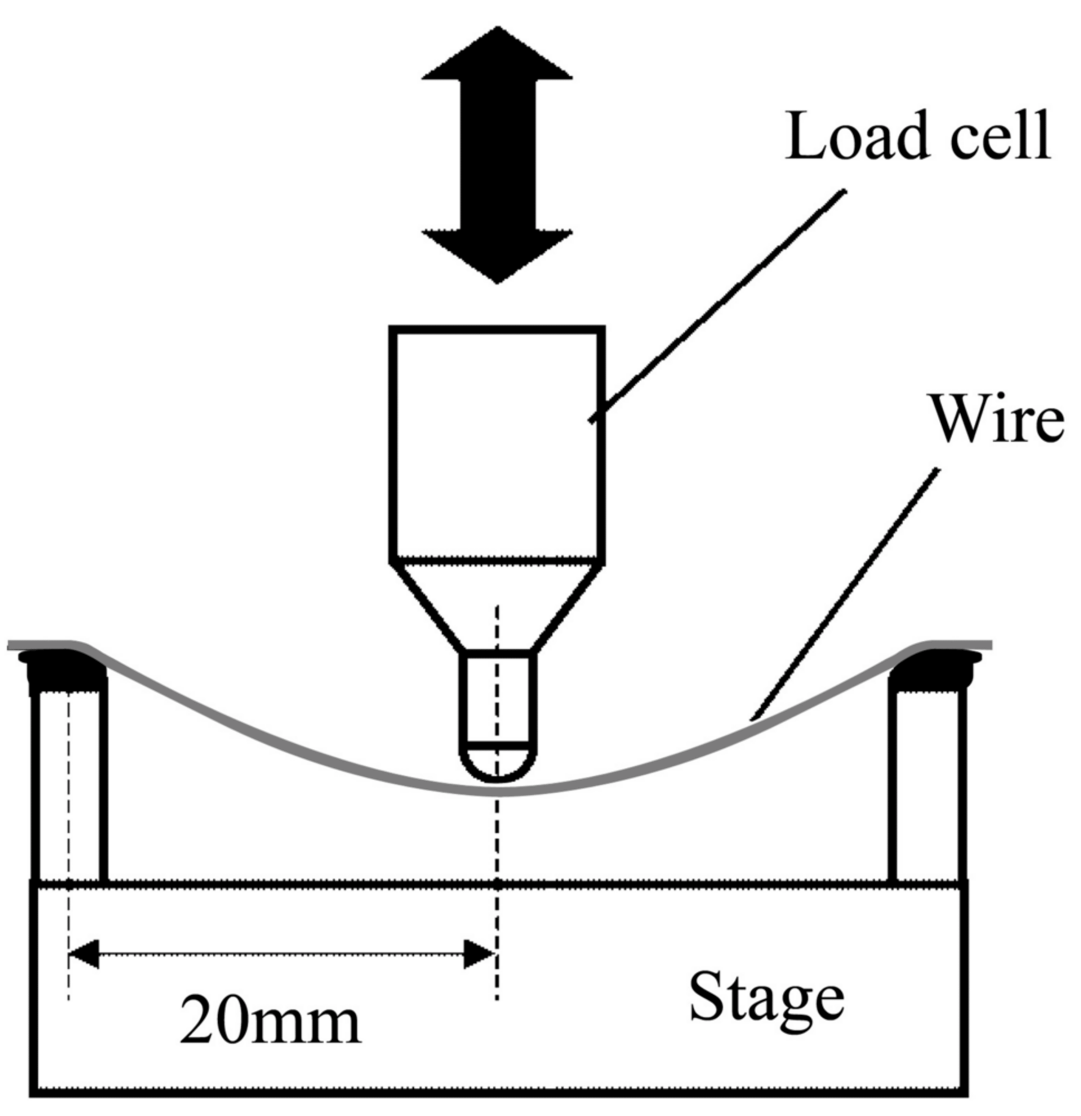
Schematic diagram of tensile test

A standard tensile test was conducted for the archwires from groups I to III using a universal testing machine (Model No. N466605; Shimadzu Corporation, Kyoto, Japan), as shown in Figure 3. Before testing, all wire specimens were inspected for surface irregularities and standardised to a uniform length. The test was performed using wire specimens of 40 mm length with a gauge length of 25 mm. Specimens were secured using mechanical wedge grips equipped with specialised flat and textured jaw faces to minimise slippage during testing. A crosshead speed of 1 mm/min was employed throughout the procedure. Testing was conducted under controlled laboratory conditions at room temperature under normal atmospheric conditions. The universal testing machine was calibrated according to internationally accepted standards, including ISO 7500-1/ASTM E4 recommendations. Calibration of the load cell was performed using high-precision reference standards through the deadweight master-cell comparison procedure prior to testing. 

During testing, tensile force was applied continuously until fracture of the specimen occurred. The maximum load sustained by the wire before fracture was recorded automatically by the machine software. UTS was then calculated by dividing the fracture load by the cross-sectional area of the wire specimen.

Strain at UTS was calculated by dividing the total extension at peak load by the original gauge length. The load deflection data generated during tensile testing were converted into stress-strain curves for each specimen, and these curves were used to determine E and YS. The observed mechanical properties were compared among the three archwire groups to evaluate inter-manufacturer differences in material behaviour.

Statistical analysis 

Data were entered into Microsoft Excel (Redmond, WA, USA) and later analysed using SPSS version 25 (IBM Corp., Armonk, NY, USA). Descriptive statistics were used to summarise the mechanical properties of the three archwire materials, including UTS, YS, and E. Normality of the data distribution was assessed using the Kolmogorov-Smirnov test and the Shapiro-Wilk test. Both tests demonstrated statistically significant p values (p < 0.05), indicating deviation from normal distribution. Since the data were not normally distributed, the results were expressed as median and interquartile range (IQR), along with minimum and maximum values. As the assumptions of normality were not satisfied, a non-parametric statistical test was employed for inferential analysis. The Kruskal-Wallis test was used to compare the mechanical properties among the three groups. Mean ranks, Kruskal-Wallis H statistics, and p values were reported. A p value less than 0.05 was considered statistically significant.

## Results

Elemental analysis 

SEM evaluation with elemental analysis demonstrated that the alloys from groups I to III were composed predominantly of iron, nickel, chromium, and carbon (Table 1). However, slight variations in the percentage composition of these elements were observed among the three archwire brands.

**Table 1:** Compositions of the three wire types. 3M Unitek (Monrovia, CA, USA), G&H Orthodontics (Franklin, IN, USA), DTech (Pune, India).

Elements	Weight (%)		
	3M Unitek	G&H	DTech
Iron	66.73	69.36	68.42
Chromium	17.77	17.83	17.83
Nickel	8.16	8.91	8.16
Carbon	7.34	3.95	5.59

**Figure 4:**
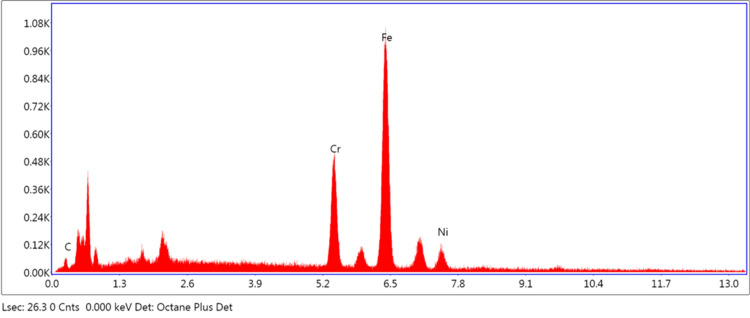
Energy-dispersive X-ray spectroscopy (EDX) spectrum of 3M showing elemental peaks. C, carbon; Cr, chromium; Fe, iron; Ni, nickel. 3M: 3M Unitek (Monrovia, CA, USA).

**Figure 5:**
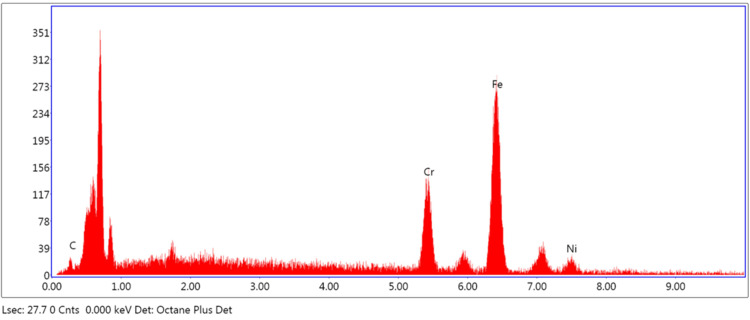
Energy-dispersive X-ray spectroscopy (EDX) spectrum of GH showing elemental peaks. C, carbon; Cr, chromium; Fe, iron; Ni, nickel. GH: G&H Orthodontics (Franklin, IN, USA)

**Figure 6:**
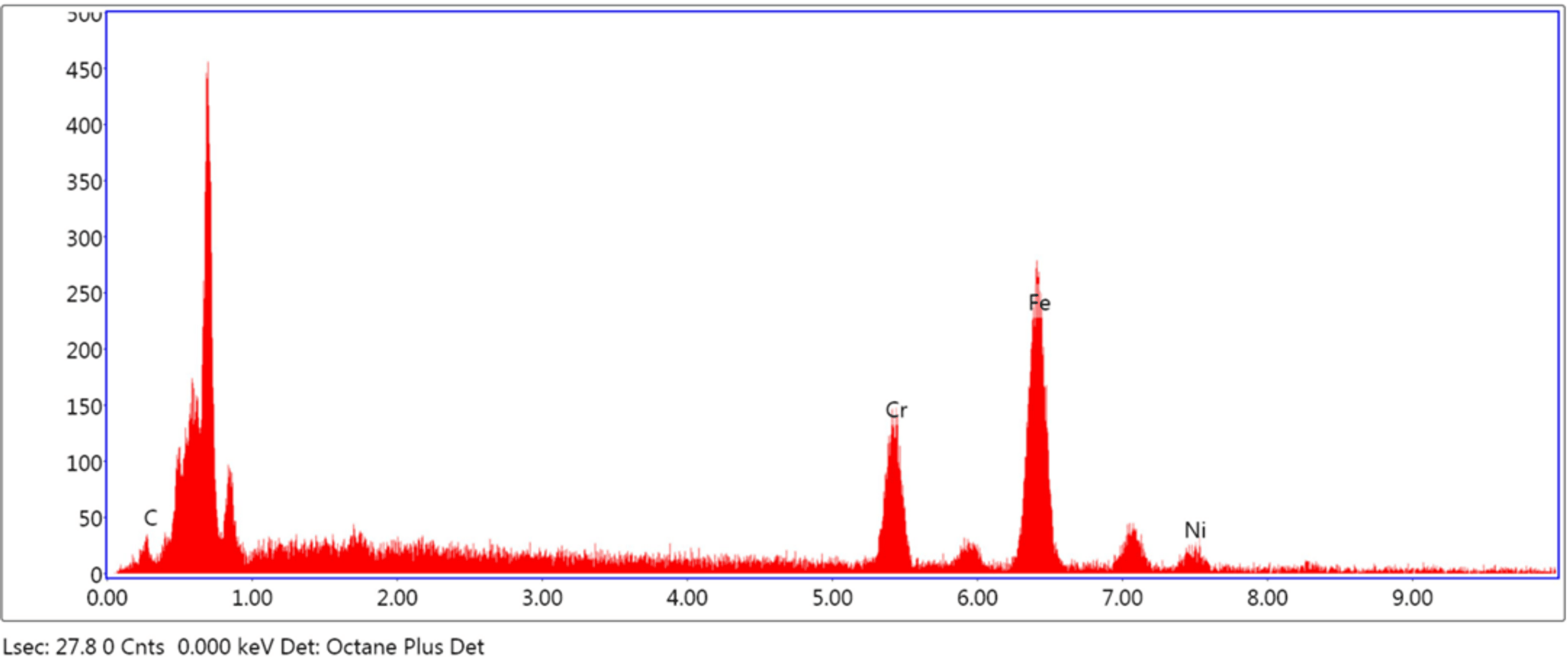
Energy-dispersive X-ray spectroscopy (EDX) spectrum of DT showing elemental peaks. C, carbon; Cr, chromium; Fe, iron; Ni, nickel. DT: DTech (Pune, India)

Among the tested groups, Group I (3M) exhibited the highest carbon content (7.34%), followed by Group III (DT) (5.59%) and Group II (GH) (3.95%). The proportions of nickel and chromium were relatively similar across all three groups. Iron content was highest in GH, followed by DT and 3M. The EDX spectrum of 3M showing elemental peaks of carbon, chromium, iron, and nickel is shown in Figure 4, the spectrum for GH is shown in Figure 5, and that for DT is depicted in Figure 6.

UTS, E, and YS

**Figure 7:**
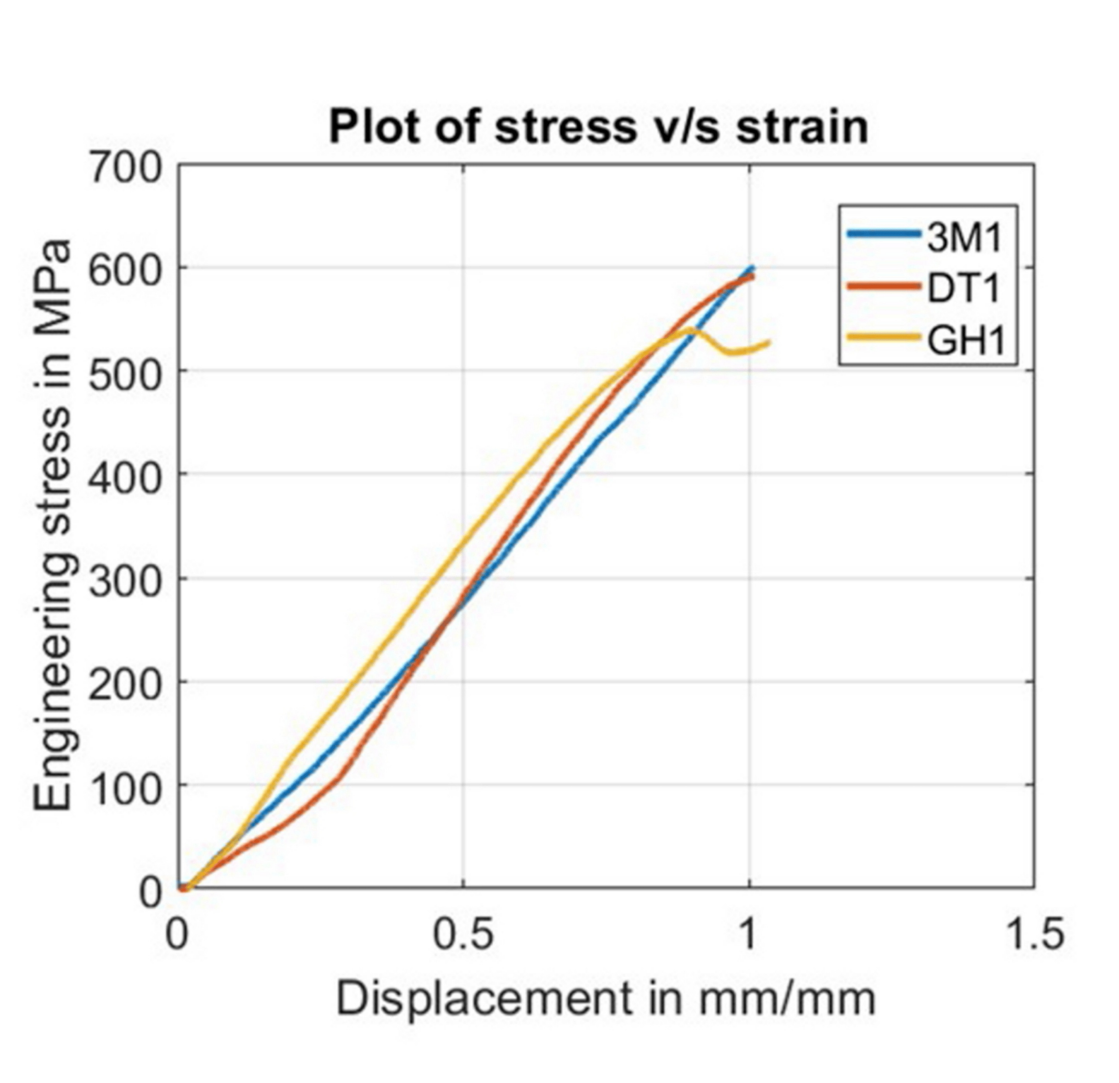
Stress strain curve for the archwires evaluated. 3M1: 3M Unitek (Monrovia, CA, USA), GH1: G&H Orthodontics (Franklin, IN, USA), DT1: DTech (Pune, India).

Load deflection curves obtained through tensile tests were plotted as stress-strain curves for each wire group, as shown in Figure 7. The Kruskal-Wallis test showed no statistically significant difference in UTS among the three groups (H = 3.837, p value = 0.147). For UTS, 3M demonstrated the highest median value (5.14), followed closely by DT (5.07), while GH showed the lowest median value (4.62). YS showed a statistically insignificant difference among the materials (H = 8.029, p-value = 0.121). 3M exhibited the highest mean rank, indicating higher YS values, while GH showed the lowest mean rank. Similarly, E also demonstrated a statistically insignificant difference among the materials (H = 5.000, p-value = 0.152). DT showed the highest mean rank, followed by GH, whereas 3M had the lowest mean rank. Overall, 3M archwires demonstrated better mechanical performance in terms of UTS and YS, but DT showed better E. However, the differences observed among the groups were statistically insignificant.

The load deflection curves obtained through tensile tests were plotted as stress-strain curves for each archwire alloy. The descriptive statistics and comparative values for UTS, YS and E of the three groups obtained through tensile testing are summarised in Tables 2, 3.

**Table 2:** Comparison of Ultimate Tensile Strength, Yield Strength, and Modulus of Elasticity Among Different Materials Using Kruskal-Wallis Test. 3M: 3M Unitek (Monrovia, CA, USA), GH: G&H Orthodontics (Franklin, IN, USA), DT: DTech (Pune, India). N, sample size. * The p-value is significant if < 0.05.

Variable	Material	N	Mean Rank	Kruskal-Wallis H	p value*
Ultimate Tensile Strength (UTS)	3M	7	13.71	3.837	0.147
	GH	7	7.43		
	DT	7	11.86		
Yield Strength	3M	7	18.00	8.029	0.121
	GH	7	4.00		
	DT	7	11.00		
Modulus of Elasticity	3M	7	4.00	5.000	0.152
	GH	7	11.00		
	DT	7	18.00		

**Table 3:** Descriptive Statistics of Ultimate Tensile Strength, Yield Strength, and Modulus of Elasticity Among Different Materials. 3M: 3M Unitek (Monrovia, CA, USA), GH: G&H Orthodontics (Franklin, IN, USA), DT: DTech (Pune, India). N, sample size. IQR, interquartile range.

Material	Variable	N	Median (IQR)	Minimum	Maximum
3M	Ultimate Tensile Strength (UTS)	7	5.14 (3.08-5.24)	3.08	5.24
	Yield Strength	7	1644.00 (1640.00-1648.00)	1640	1648
	Modulus of Elasticity	7	200.40 (200.40-200.40)	200.40	200.40
GH	Ultimate Tensile Strength (UTS)	7	4.62 (3.43-4.88)	3.43	4.88
	Yield Strength	7	1609.00 (1605.00-1610.00)	1605	1610
	Modulus of Elasticity	7	201.20 (201.20-201.20)	201.20	201.20
DT	Ultimate Tensile Strength (UTS)	7	5.07 (4.17-5.27)	4.17	5.27
	Yield Strength	7	1662.00 (1651.00-1670.00)	1651	1670
	Modulus of Elasticity	7	201.80 (201.80-201.80)	201.80	201.80

## Discussion

SS archwires remain the most commonly used working and retraction archwires in orthodontic practice because of their high rigidity and relatively low frictional characteristics during sliding mechanics [1]. In contrast, more flexible archwires may undergo greater deformation under orthodontic loading, potentially resulting in undesirable effects such as archwire bowing, roller-coaster effects, increased binding, and reduced efficiency of tooth movement [6].

SS archwires are manufactured by multiple companies under different brand names. However, material composition and the mechanical properties of these products are not routinely disclosed by manufacturers and may not be uniform across brands. Clinically, orthodontists often perceive variations in rigidity and deflection behaviour among wires from different manufacturers. 

Most studies have primarily evaluated the mechanical behaviour of SS archwires with round cross sections or 0.017 x 0.025-inch dimensions [7-10]. To date, limited information is available regarding the comparison of 0.019 x 0.025-inch SS archwires from different commercially available brands. Therefore, the present study was undertaken to evaluate and compare the mechanical properties and elemental composition of three commonly used brands representing different economic tiers in the market: 3M Unitek, G&H Orthodontics, and DTech. 

The elemental analysis demonstrated compositions broadly comparable to those reported by Brantley and Eliades [3], except for the carbon content among the groups. Carbon concentration was highest in 3M, which could potentially make the alloy brittle. However, comparison of the UTS revealed no statistically significant differences among the groups. Carbon content exceeding 0.2% may alter the metallurgical characteristics of SS toward carbon steel, thereby questioning the corrosion resistance properties of the wire. Carbon content in carbon steel increases hardness but makes the alloy brittle [3]. Nevertheless, SS generally exhibits good corrosion resistance intraorally, although soldered joints are more susceptible to corrosion in the oral environment [1]. The nickel and chromium contents in all groups were found to be close to the ideal 18:8 composition considered suitable for orthodontic applications.

Among the various mechanical tests available to engineers, UTS is one of the most commonly employed parameters and serves as a routine benchmark for product development and quality control, although it may not always represent the most clinically relevant property [2]. UTS reflects the fracture resistance of the wire, which is clinically relevant, especially during the applications of loop mechanics [2,8]. Comparison of UTS values showed the highest values in 3M, followed by DT and GH; however, the differences were statistically insignificant. These values for SS archwires are similar to the studies conducted by Krishnan and Kumar [8] and Juvvadi et al. [9]. However, they did not evaluate inter-manufacturer wire differences. While orthodontists do not directly utilise UTS values in clinical practice, comparative evaluation of these data provides useful insight into the mechanical performance and fracture resistance characteristics of orthodontic archwires.

E is the inherent stiffness of the material, which is the ratio of unit stress to unit strain. As the same alloy was used in all three groups in the present study, E would be expected to remain essentially constant among the groups. However, there was some statistically insignificant difference among the groups. It was higher in Group DT, followed by GH and 3M. Brantly and Eliades [3] reported a high flexural modulus of SS of approximately 36x106 psi, with variations in measured values being attributed primarily to differences in specimen length. But in our study length was standardised across all groups; therefore, the observed differences could be attributed to minor variations in composition among the groups. 

The difference in E among the groups in the present study may be attributed to microstructural changes induced by extensive cold working, including lattice distortion, residual internal strains, and preferred crystallographic orientation, all of which can influence the mechanical characteristics of the alloy. Goldberg et al. also suggested that the differences in modulus, reaching nearly 20%, may occur as a consequence of severe cold drawing during the manufacturing process [11]. However, Kusy and Dilley [12] proposed that the comparatively lower elastic modulus values may not be necessarily related to the degree of cold working but instead associated with methodological challenges encountered during mechanical testing of wires with very small cross-sectional dimensions. Wires with higher E resist deflection under applied forces and are therefore more suitable during the retraction phase, particularly in cases requiring control of the occlusal plane [6].

YS denotes the proportional limit, which is the point beyond which increasing force produces permanent deformation [3]. In the present study, 3M exhibited the highest YS among the three groups, though the difference was not statistically significant. These values are marginally higher than those reported by Krishnan and Kumar [8] and Juvvadi et al. [9], which may be attributable to their use of 0.017 x 0.025-inch SS wires. When two wires have similar stiffness but different yield strengths, the one with the higher YS is preferable [2]. In addition to reducing clinical issues that can distort the archwire, a greater YS expands the working range and enhances the ability to store potential energy. Therefore, 3M can store the most potential energy and undergoes the least deformation among the three groups. 

Limitations of the study

An extensometer could not be used because attachment of it to the wire resulted in slippage. The absence of an extensometer means that strain measurement accounts for both machine compliance and sample strain. The Rp0.2 method requires precise strain measurement at 0.2%, which cannot be ensured in this case. Since the linear region cannot be identified with confidence, it was hard to find the exact values. Future studies employing tensile tests with extensometers can accurately predict the strain measurement. Carbon and other light elements were evaluated using standard matrix correction algorithms; however, due to the inherent limitations of EDX in accurately quantifying light elements and the potential for surface hydrocarbon contamination, carbon percentage should be assessed with caution. Sample size was also limited, which may limit the generalizability of the findings to other wire dimensions, production batches, and manufacturers.

## Conclusions

The results demonstrated that orthodontic wires are no anomalies; they either meet or exceed the specifications. During elemental analysis, considerable variation in carbon percentage was observed among the three groups, in descending order: 3M Unitek (7.34%), DTech (5.59%), and G&H Orthodontics (3.95%). The corrosion resistance of commercially available SS archwires may not be consistent with the expected material properties because of the marked variation in carbon content among brands. These findings necessitate clinical vigilance during treatment, including periodic evaluation of retraction wires for surface deterioration, corrosion, or deformation. UTS was highest in 3M Unitek, followed by DTech and G&H Orthodontics, although the differences were statistically insignificant. 3M Unitek had the highest YS (statistically insignificant) among the three groups, showing the least chance of permanent deformation. DTech had the stiffest wire (E), although statistically insignificant, making it more efficient in resisting archwire deflection, particularly in cases requiring occlusal plane control. Although numerical differences were observed but were not statistically significant, further studies using larger samples and clinically relevant mechanical and corrosion tests are needed before making clinical recommendations and proper brand comparisons.

## References

[1] Kapila S, Sachdeva R (1989). Mechanical properties and clinical applications of orthodontic wires. Am J Orthod Dentofac Orthop.

[2] Kusy RP, Dilley GJ, Whitley JQ (1988). Mechanical properties of stainless steel orthodontic archwires. Clin Mater.

[3] Brantley WA, Eliades T (2000). Orthodontic Materials: Scientific and Clinical Aspects. Orthodontic Materials: Scientific and Clinical Aspects. Georg.

[4] (1982). Engineering Properties of Steel. Metals.

[5] Kusy RP, Greenberg AR (1981). Effects of composition and cross section on the elastic properties of orthodontic wires. Angle Orthod.

[6] Yang WS, Kim BH, Kim YH (2001). A study of the regional load deflection rate of multiloop edgewise arch wire. Angle Orthod.

[7] Burstone CJ, Goldberg AJ (1983). Maximum forces and deflections from orthodontic appliances. Am J Orthod.

[8] Krishnan V, Kumar KJ (2004). Mechanical properties and surface characteristics of three archwire alloys. Angle Orthod.

[9] Juvvadi SR, Kailasam V, Padmanabhan S, Chitharanjan AB (2010). Physical, mechanical, and flexural properties of 3 orthodontic wires: an in-vitro study. Am J Orthod Dentofacial Orthop.

[10] Yoshikawa DK, Burstone CJ, Goldberg AJ, Morton J (1981). Flexure modulus of orthodontic stainless steel wires. J Dent Res.

[11] Goldberg AJ, Morton J, Burstone CJ (1983). The flexure modulus of elasticity of orthodontic wires. J Dent Res.

[12] Kusy RP, Dilley GJ (1984). Elastic modulus of a triple-stranded stainless steel arch wire via three- and four-point bending. J Dent Res.

